# Composition of Flavonoids in the Petals of *Freesia* and Prediction of Four Novel Transcription Factors Involving in *Freesia* Flavonoid Pathway

**DOI:** 10.3389/fpls.2021.756300

**Published:** 2021-11-15

**Authors:** Jiayi Zhu, Xueying Guo, Xin Li, Dongqin Tang

**Affiliations:** ^1^School of Design, Shanghai Jiao Tong University, Shanghai, China; ^2^Instrumental Analysis Center, Shanghai Jiao Tong University, Shanghai, China

**Keywords:** *Freesia hybrida*, flavonoid, transcription factor, WRKY, AP2

## Abstract

*Freesia hybrida* is rich in flower colors with beautiful flower shapes and pleasant aroma. Flavonoids are vital to the color formation of its flowers. In this study, five *Freesia* cultivars with different flower colors were used to study on the level of accumulation of their flavonoids and expression of flavonoid-related genes and further explore new novel transcription factor (TF). Ultra-high-performance liquid chromatography and VION ion mobility quadrupole time-of-flight mass spectrometer (UPLC-Q-TOF-MS) were used to determine the flavonoids. Combined with transcriptome sequencing technology, the molecular mechanism of the flavonoid metabolism difference in *Freesia* was revealed. A total of 10 anthoxanthin components and 12 anthocyanin components were detected using UPLC-Q-TOF-MS. All six common anthocyanin aglycones in high plants, including cyanidin, delphinidin, petunidin, peonidin, malvidin, and pelargonidin, were detected in *Freesia* at first time in this study. In orange, yellow, and white cultivars, anthoxanthins gradually decreased with the opening of the petals, while in red and purple cultivars, anthoxanthins first increased and then decreased. No anthocyanin was detected in yellow and white cultivars, while anthocyanins increased with the opening of the petals and reached their maximum at the flowering stage (S3) in other three cultivars. The correlation analysis revealed that the color of *Freesia* petals was closely related to the composition and content of anthoxanthins and anthocyanins. Petals of five cultivars at S3 were then selected for transcriptome sequencing by using the Illumina Hiseq 4000 platform, and a total of 100,539 unigenes were obtained. There were totally 5,162 differentially expressed genes (DEGs) when the four colored cultivars were compared with the white cultivar at S3. Comparing all DEGs with gene ontology (GO), KEGG, and Pfam databases, it was found that the genes involved in the flavonoid biosynthesis pathway were significantly different. In addition, AP2, WRKY, and bHLH TF families ranked the top three among all differently expressed TFs in all DEGs. Quantitative real-time PCR (qRT-PCR) technology was used to analyze the expression patterns of the structural genes of flavonoid biosynthesis pathway in *Freesia*. The results showed that metabolic process was affected significantly by structural genes in this pathway, such as *CHS1*, *CHI2*, *DFR1*, *ANS1*, *3GT1*, and *FLS1*. Cluster analysis was performed by using all annotated WRKY and AP2 TFs and the above structural genes based on their relatively expression. Four novel candidate TFs of WRKY and AP2 family were screened. Their spatiotemporal expression patterns revealed that these four novel TFs may participate in the regulation of the flavonoid biosynthesis, thus controlling its color formation in *Freesia* petals.

## Introduction

Plant pigments mainly include flavonoids, carotenoids, and betalain substances. Among them, flavonoids, an important kind of plant secondary metabolites, are most widely distributed in plants (Mierziak et al., 2014; Nabavi et al., 2020), and they can be divided into flavonols, flavones, anthocyanins, proanthocyanidins, and catechins, etc., based on the degree of oxidation and conformational differences of their three-carbon bonds (Singh et al., 2014). Commonly, flavonoids and flavonols are collectively called anthoxanthins (Kesavan et al., 2018).

Flavonoids have been proved to affect the coloration of many plants, which can make plants appear milky white, yellow, orange-red, blue-violet, and other colors and have a decisive effect on the color of a variety of plant fruits, leaves, and petals. For instance, a significant correlation was observed between the hue, brightness, and vividness of the petals of *Narcissus* cultivars and its flavonoids (Li et al., 2015); 29 anthocyanins were identified in iris, among which delphinidin contributes to the formation of the flower color of purple wild iris (*Iris dichotoma*), while the flower color of orange *I*. *domestica* is mainly determined by pelargonidin (Xu et al., 2018). In *Rhododendron* species, anthocyanins, such as delphinidin and cyanidin, and flavonols, such as quercetin and kaempferol, were detected in the petals. It was also found that anthocyanins promote the formation of red flowers of *Rhododendron* (Du et al., 2018).

Structural genes in the flavonoid biosynthesis pathway, including *CHS*, *CHI*, *F3H*, *F3′5′H*, *FNS*, *FLS*, *DFR*, *ANS*, and *UF3GT*, encode key enzymes to control the synthesis of corresponding flavonoids, thereby affecting the color development of petals. For instance, the *CHS* gene of *Hosta plantaginea* was introduced into tobacco (*Nicotiana tabacum*), which deepened the flower color of transgenic plants and significantly increased the flavonoid content (Zhang et al., 2020). In *Rosa hybrida*, the expression of *F3′5′H* gene led to the accumulation of delphinidin, which made the petals appear in a novel bluish flower color (Katsumoto et al., 2007). The *FLS* gene of *Rosa rugosa* was introduced into petunia, which made some petunia plants show a lighter flower color, and a new flavonol myricetin was detected at the same time (Tsuda et al., 2004). The *FLS* gene was antisensely expressed in *Eustoma grandiflorum*, which turned its flower color into magenta. The transgenic plants also contain new anthocyanin components and accumulate more dihydroflavonols (Nielsen et al., 2002). *DFR* and *ANS* are essential for the synthesis of anthocyanins. In tulip petals, it was suggested that the accumulation of the red pigment was positively related to the expression levels of *TfDFR1* and *TfANS1* (Yuan et al., 2013). Chen M. et al. (2018) found that the functional defect or mutation of the *ANS* gene may be the cause of the white flower cultivar of *Solanum melongena*. In *Freesia*, some members in an individual structural gene family, including *FhFLS1* and *FhFLS2* (Shan et al., 2020), *FhCHS1* (Sun et al., 2015), *FhDFR*s (Li et al., 2017), and *Fh3GT*s (Sun et al., 2016; Meng et al., 2019), were also isolated, which were confirmed to be related with the flavonoid biosynthesis so far.

Till present, various regulatory genes that directly control transcription of the structural genes involving in the flavonoid biosynthesis pathway had been identified from some plants. Among them, three transcription factors (TFs) families, namely, R2R3-MYB, basic helix-loop-helix (bHLH), and WD40 repeats (WD40), have been extensively studied. They can regulate the structural genes involving in the flavonoid pathway alone or in the form of MBW complex (Xu et al., 2015; Chen et al., 2019; Yuan et al., 2020). In *Freesia*, several reports focused on these three TFs, and their regulating mechanism in this pathway is also relatively clear (Li et al., 2016, 2019, 2020; Shan et al., 2020). With the discovery of more powerful TF families, scientists are also paying attention to whether there are other novel TFs other than the above three families, involving in the regulation of the flavonoid biosynthesis in a certain plant.

*Freesia*, a perennial herb belonging to the genus *Freesia* in the Iridaceae family, is native to southern Africa (Manning and Goldblatt, 2010). *Freesia hybrida* is a collective name for many horticultural cultivars in this genus and currently planted worldwide as a cut flower with rich colors and pleasant fragrance. The main colors of the petals in *F*. *hybrida* include white, yellow, red, and purple. It was already confirmed that flavonoids had a decisive effect on the color of *Freesia* flower (Zhong et al., 2009). Our previous study determined various flavonoid components in *Freesia* petals and found that the petal color of *F*. *hybrida* was related with the flavonoid components; meanwhile, the degree of petal coloration was proportional to the total amount of flavonoids in *Freesia* petals (Xu et al., 2016; Yu et al., 2020; Zhu et al., 2021). These results provide a good foundation to further study the mechanism of formation of flower color in *Freesia* on the basis of the metabolic and molecular levels.

## Materials and Methods

### Plant Materials and Growth Conditions

**FIGURE 1:**
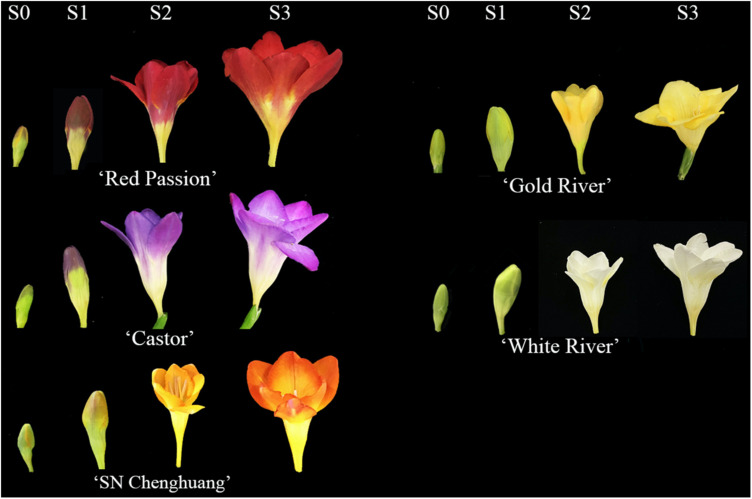
Flower color phenotypes of five *Freesia hybrida* cultivars at different developmental stages.

Five cultivars of *F*. *hybrida* with different colors were used as materials, namely, ‘Castor’ (purple), ‘SN Chenghuang’ (orange), ‘Gold River’ (yellow), ‘Red Passion’ (red), and ‘White River’ (white) (Figure 1), shortened as CA, CH, GR, RP, and WR in the following text, figures, and tables, respectively.

The healthy corms of the above cultivars were planted in the standard farm shed at the Modern Agricultural Engineering Training Center of Shanghai Jiao Tong University in late October 2019, and the petal samples were collected at four developmental stages (S0–S3) of each cultivar during March to April, 2020. The description of each stage is presented in Supplementary Table 1.

### Identification and Measurement of the Flavonol and Anthocyanin

Ultra-high-performance liquid chromatography and VION ion mobility quadrupole time-of-flight mass spectrometer (UPLC-Q-TOF-MS) (Waters Corporation, United States) with Optima LC/MS-grade methanol and trifluoroacetic acid (Thermo Fisher Scientific, United States) were used to identify flavonol and anthocyanin and measure their contents in petals of *Freesia*.

Several standards were used for the qualitative and quantitative identification of anthocyanins and anthoxanthins in *Freesia* petals. All standards are shown in Supplementary Table 2.

Ultra-high-performance liquid chromatography and VION ion mobility quadrupole time-of-flight mass spectrometer analysis conditions are as follows: The column is Ethylidene bridge hybrid particles (BEH) C18 1.7 μm (2.1 mm × 100 mm); mobile phase A, 0.1% formic acid H_2_O; B, 0.1% formic acid acetonitrile. The elution gradient is 0 min, 5% B; 3 min, 20% B; 10 min, 100% B; 12 min, 100% B; 15 min, 95% B; 20 min, 95% B. The flow rate is 0.4 mL/min. The injection volume is 1 μl and the column temperature is 45°C. Mass spectrometry conditions are as follows: Acquisition mode is MSE (low-energy/high-energy switching scan); anthoxanthin detection ion mode is electrospray negative ion scan mode (*m*/*z* 50–1,000), anthocyanin is electrospray positive ion scan mode (*m*/*z* 50–1,000), the scanning speed is 0.2 s. The capillary voltage is 2 kV. The cone voltage is 40 V. The temperature of the atomizing gas is 450°C. The flow of atomizing gas is 900 L/h. The taper hole blowback gas is 50 L/h. The ion source temperature is 115°C.

The flavonoids in petals of *Freesia* were quantified by using the external standard method. The sum of the content of each anthoxanthin component was calculated and recorded as the total anthoxanthin content, that is, the total flavone and flavonol content [total flavonoid content (TFC)]; the sum of the content of each anthocyanin component was calculated and recorded as the total anthocynadin content (TAC).

### RNA Extraction and Transcriptome Sequencing

The petals at S3 of each cultivar were then chosen as materials for transcription sequencing. Three biological duplicate samples were taken from each cultivar.

RNA of each sample was extracted using TRIzol Reagent (Kangwei Century Ltd., Beijing, China). The RNA quality test uses Nanodrop GX. Biomarker Technologies (Beijing, China) was responsible for transcriptome sequencing. The Illumina Hiseq 4000 platform was used for sequencing, and a large number of high-quality reads (raw data) were produced. Then, the clean data were obtained by filtering these raw data, discarding low-quality reads and the connector sequence.

### *De novo* Transcriptome Assembling, Functional Annotation, and Classification of Unigenes

Trinity software^1^ was used to assemble the obtained clean data. First, the reads were broken into shorter fragments (K-mer), then these small fragments were extended into longer fragments (Contig), and the overlap between these fragments was used to obtain a fragment set (component). Finally, using the method of De Bruijn diagram and the sequencing read information, the transcripts were identified in each fragment set to obtain the unigene library of *Freesia* petals. The sequencing data have been uploaded to the NCBI database (BioProject: PRJNA656641).

The alignment of Freesia unigene sequences with gene ontology (GO)^2^, KEGG^3^, COG^4^, Pfam^5^, and other eight databases was conducted by using BLAST. The prediction of unigene amino acid sequence was performed by using HMMER^6^, and the KEGG Orthology analysis is conducted by using KOBAS2.0^7^.

### Differential Expression Analysis of Unigenes

Bowtie was used to compare the read length obtained by sequencing with the unigene library and to estimate the expression level combined with RNA-Seq by Expectation-Maximization. Fragments per kilobase per million (FPKM) was used to indicate the expression abundance of the corresponding unigene.

DESeq2 is used for the differential expression analysis of genes between sample groups. The screening criteria are fold change (FC) ≥ 2 and false discovery rate (FDR) < 0.01. Subsequently, the differentially expressed gene (DEG) sets were obtained, named in the form of “A vs. B.”

### Screening of Key Genes in Flavonoid Biosynthesis Pathway

The transcriptome database was searched for key structural genes of the flavonoid biosynthesis pathway based on the functional annotation, and the selected genes were further confirmed by comparing with the known genes in the NCBI database through BLAST+. Genes with high matching degree and high expression abundance (FPKM) were selected for following validation by quantitative real-time PCR (qRT-PCR) detection.

The WRKY, AP2 family TFs were preliminarily screened from the transcriptome according to the functional annotation and then were used to cluster with the above selected structural genes. These TFs closer to the structural genes were selected as candidates. According to the relative gene expression multiples between samples, the software Multi Experience Viewer 4.9.0^8^ was used for the gene cluster analysis and heat map drawing. Furthermore, the transcriptome data (BioProject: PRJNA656641) of *Freesia* corms measured by our group were compared, and TFs with high expression in petals and low expression in corms were chosen to perform the phylogenetic analysis and study their temporal and spatial expression characteristics *via* qRT-PCR. The gene phylogenetic tree was constructed by using the MEGA-X software, and the phylogenetic tree was constructed by neighbor-joining method with a step value of 1,000. The red cultivar RP with high flower pigments was used to conduct qRT-PCR, and the roots, stems, leaves, and petals at four developmental stages were sampled, respectively.

### The Transcript Level Analysis of Flavonoid-Related Gene by Quantitative Real-Time PCR

The sequences of the selected genes were used to design primers by using the Primer 5.0 software^9^. Actin was used as the internal reference gene (Ding et al., 2020). All primer sequences are listed in Supplementary Table 3. The primers were synthesized by Tsingke Biotechnology Co., Ltd. (Shanghai, China).

The first-strand cDNA was obtained by using Prime Script^TM^ RT reagent kit (TaKaRa, Dalian, China) and then used as the template for qRT-PCR. A total 20-μl PCR reaction system was prepared, including 10 μl of SYBR, 7.2 μl of ddH_2_O, 0.4 μl of each forward primer and reverse primer, and 2.0 μl of cDNA. The reaction program was set to 94°C for 10 min, followed by 40 cycles with 94°C for 20 s, 55°C for 20 s, and 72°C for 20 s.

The relative expression amount of the selected genes was calculated according to the 2^–△△Ct^ method (Livak and Schmittgen, 2001). The sample with the lowest expression level was taken as the calibrator for each gene, and it was set to 1.

### Statistical Analysis

Three biological replicates were included for the measurement of the anthoxanthin and anthocyanin and qRT-PCR for each gene. In figures, the values were present as the mean ± standard error (SE) of three replicates. Significance was determined by Duncan test (*p* < 0.05) by SPSS Statistics version 19.0.

## Results

### Analysis of Flavonoid Accumulations in *Freesia* Flowers

Eight flavonol components and two dihydroflavonoid components were detected in the petals of *Freesia* by UPLC-Q-TOF-MS (Table 1). Among them, component 1, component 2, component 3, and component 4 were identified through the standard chemicals, which were kaempferol, quercetin, quercetin-3-*O*-rutinoside, and quercetin 3-galactoside, respectively. The other six components were inferred based on the mass spectrum data.

**TABLE 1:** Identification of anthoxanthins component from *Freesia hybrida* petals.

**Component**	**Retention time (min)**	**Quasi-molecular ions (m/z)**	**Fragment ions (m/z)**	**Tentative identification**
1	10.62	285	151	Kaempferol*
2	9.13	301	107	Quercetin*
3	5.76	609	301	Quercetin 3-*O* rutinoside*
4	5.93	463	301	Quercetin 3-galactoside*
5	6.13	623	315	Isorhammetin-R
6	6.09	463	301	Quercetin-G
7	5.48	609	301	Quercetin-N
8	6.85	447	285	Kaempferol-G
9	5.71	433	271	Naringenin-G
10	6.93	447	271	Naringenin-Gr

*Stands for compounds identified by standards; G, glucoside; Gr, glucuronide; R, rutinoside; and N, neohesperidoside.

The anthocyanin components of *Freesia* petals were identified, and 12 anthocyanin components were obtained (Table 2). According to the standards, six components, namely, component 1, component 2, component 3, component 4, component 5, and component 6, were identified exactly, which are cyanidin 3-*O*-glucoside, delphinidin-3-*O*-glucoside, petunidin 3-*O*-glucoside, peonidin 3-*O*-glucoside, malvidin 3-*O*-glucoside, and pelargonidin 3-*O*-glucoside, respectively. The other six components were inferred based on the mass spectrum data.

**TABLE 2:** Identification of anthocyanins component from *Freesia hybrida* petals.

**Component**	**Retention time (min)**	**Quasi-molecular ions (m/z)**	**Fragment ions (m/z)**	**Tentative identification**
1	5.46	449	287	Cyanidin 3-*O*-glucoside*
2	4.98	465	303	Delphinidin 3-*O*-glucoside*
3	5.80	479	317	Petunidin 3-*O*-glucoside*
4	6.31	463	301	Peonidin 3-*O*-glucoside*
5	6.56	493	331	Malvidin 3-*O*-glucoside*
6	5.94	433	271	Pelargonidin 3-*O*-glucoside*
7	4.32	773	287	Cyanidin-GGG
8	4.25	627	303	Delphinidin-GG
9	4.71	611	303	Delphinidin-R
10	4.95	641	317	Petunidin-GG
11	5.48	625	301	Peonidin-GG
12	5.65	655	331	Malvidin-GG

*Stands for compounds identified by standards; G, glucoside; GG, diglucoside; GGG, triglucoside; and R, rutinoside.

**FIGURE 2:**
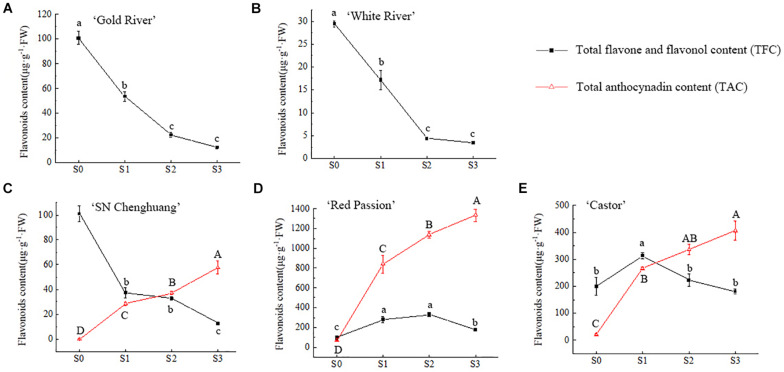
Changes of total flavonoid content (TFC) and total anthocynadin content (TAC) in four flower developing stages of ‘Gold River’ **(A)**, ‘White River’ **(B)**, ‘SN Chenghuang’ **(C)**, ‘Red Passion’ **(D)**, and ‘Castor’ **(E)** petals. Uppercase and lowercase letters stand for significance of TAC and TFC in different stages, respectively. The significance of difference is at 0.05 level measured by using the Duncan test.

The relative quantification of TFC and TAC in petals at different developmental stages was carried out and the results were shown in Figure 2.

For anthoxanthin, the TFC of GR, WR, and CH (Figures 2A–C) reached the highest value in S0 and decreased with the opening of the petals significantly (*p* < 0.05). The TFC of RP and CA (Figures 2D,E) showed an increase and then a decline, reaching the highest value at S2 and S1, respectively. At S0, except for WR (Figure 2B), TFC of all other four cultivars showed a higher level of accumulation. From S1 to S3, the TFC of RP and CA (Figures 2D,E) was significantly higher than other cultivars at each stage (*p* < 0.05), especially RP.

For anthocyanins, TAC in the petals of CH, RP, and CA (Figures 2C–E) showed a significant upward trend (*p* < 0.05) with the opening of the petals, reaching the highest value at S3. Among them, the TAC of RP (Figure 2D) was significantly higher than the other cultivars (*p* < 0.05), reaching 1,334.09 μg/g FW at S3, followed by CA (Figure 2E), reaching 406.26 μg/g FW. The lowest TAC was observed in CH, corresponding to 57.38 μg/g FW (Figure 2C). Since anthocyanins were not detected in GR and WR (Figures 2A,B), they are not shown in the figure.

### *De novo* Transcriptome Assembly and Functional Annotation of Unigenes

Since a significant difference was present in the accumulation of flavonoid among five *Freesia* cultivars at S3, petals at this stage of each cultivar were chosen as materials for transcription sequencing. Sequencing the petals of the five *Freesia* cultivars at S3, a total of 188.29 Gb clean data were obtained, and the clean data of each sample reached 4.59 Gb. After assembly, a total of 1,00,539 unigenes were obtained with a mean length of 1,126 nt. The N50 of unigene was 1,523 nt, and 24,917 unigenes were longer than 1,000 nt, showing a high assembly integrity. Other detailed data are shown in Supplementary Table 4.

Comparing the obtained unigenes with COG, GO, KEGG, and other eight databases, a total of 30,857 unigenes were annotated. The specific statistical results are shown in Supplementary Table 5.

### Differential Expression Analysis of Unigenes

The transcriptome library of petal samples of four colored cultivars at S3 was compared with that of white cultivar (WR) at S3. A total of 5,162 DEGs were obtained from the four DEG sets (Supplementary Table 6).

The largest number of DEGs of 2,584 was present in CH3 vs. WR3, followed by 2,546 in RP3 vs. WR3. The least number of DEGs was observed in GR3 vs. WR3, with only 14 genes. Comparing WR3 with the other individual cultivars, it was found that there were more downregulated genes than the upregulated genes. A total of 1,545 genes were downregulated in WR3 compared with CH3. Comparing WR3 with CA3 and RP3, 1,191 and 1,307 genes were downregulated, respectively, while only eight genes were downregulated in GR3.

Comparing the 5,162 DEGs with the GO database, a total of 2,067 DEGs were annotated (Supplementary Figure 1). Among them, a large number of DEGs were enriched in the metabolic process, cell, and catalytic activity.

Comparing all above DEGs with the KEGG database, a total of 714 genes were annotated. Among them, the flavonoid biosynthesis pathway had the highest enrichment factor of 3.52 (Supplementary Figure 2), and 22 differential genes in this pathway were annotated. The comparison of these 22 DEGs based on FPKM values indicates that their expression differs among different flower-colored cultivars of *Freesia*, and a higher expression level was present in the dark-colored cultivars than in the light-colored cultivars. Thus, the expression of genes in the flavonoids biosynthesis pathway may be closely related to the color difference of *Freesia*.

Comparing the 5,162 DEGs with the Pfam database, the top 20 TF families with the largest number were counted (Supplementary Figure 3). Among them, *AP2*, *WRKY*, and *bHLH* were ranked as the top three TF families in *Freesia*. The largest number (15 in total) was present in the *AP2* family, followed by the *WRKY* family, 13 in total, and 10 in the *bHLH* family. It suggests that the expression of *AP2*, *bHLH*, and *WRKY* TFs is significantly different among these different cultivars at S3 and may be involved in the regulation of formation of flower color in *Freesia*.

### Validation of Differential Expression of Structural Genes Involving in Flavonoid Biosynthesis by Quantitative Real-Time PCR

**FIGURE 3:**
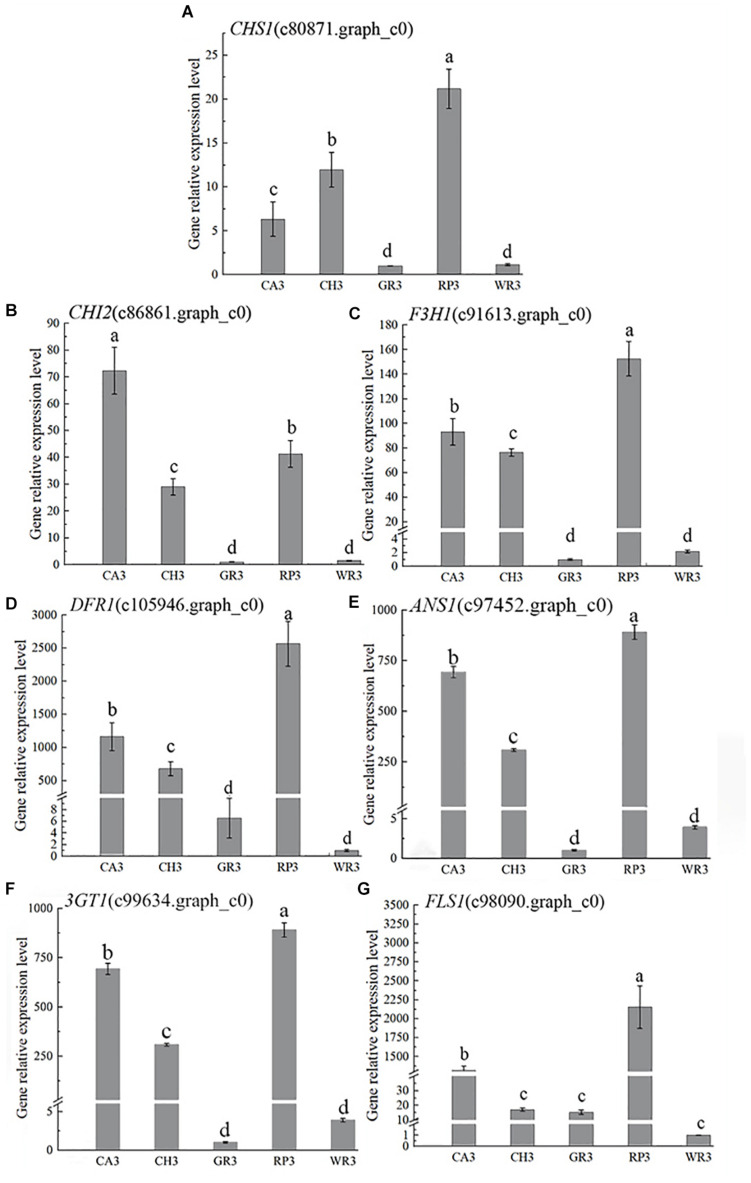
The expression characteristics of seven structural genes of *Freesia hybrida*. **(A)** *CHS1*, **(B)** *CHI2*, **(C)** *F3H1*, **(D)** *DFR1*, **(E)** *ANS1*, **(F)** *3GT1*, and **(G)** *FLS1*. GR3 was chosen as calibrator sample of *CHS1*, *CHI2*, *F3H1*, and *3GT1*; WR3 was chosen as calibrator sample of *DFR1*, *ANS1*, and *FLS1*. Lowercase letters stand for the significance of difference at 0.05 level.

Based on the above results of the flavonoid components, the structural genes in this pathway were preliminarily searched in the transcriptome database by keywords of the components. Then, BLAST+ was used to compare the related genes in the flower transcriptome database with the known genes in the NCBI database, and 13 structural genes involving in the flavonoid synthesis pathway were screened with the higher matching degree. The gene number and nomenclature are shown in Supplementary Table 7. Notably, 7 out of above 13 genes were further verified by using qRT-PCR, and their expression patterns were analyzed (Figure 3).

In the light-colored *Freesia* cultivars (GR and WR), the expression levels of all selected seven structural genes were much lower than those in the dark-colored cultivars, RP, CA, and RP, at S3. The highest expression level of *CHS1*, *CHI2*, and *F3H1* was observed in RP3, CA3, and RP3, respectively, while the expression of these genes was much lower in GR3 and WR3. The expression of *DFR1* and *ANS1* was also high in the dark-colored cultivars, peaking in RP3 and CA3, respectively, which was much higher than that in the light-colored cultivars GR and WR, consistent with no anthocyanins detected in the two cultivars. The expression pattern of *3GT1* was similar to that of *CHI2*. The highest and the lowest expressions were observed in RP3 and WR3, respectively. Although similar expression pattern of *FLS1* with *DFR1* was present, the expression level of this gene was quite different. A significantly higher level of *FLS1* was detected in RP3 than in other three cultivars.

### Explore Novel Candidate Transcription Factors Involving in Flavonoid Biosynthesis

Recently, it was confirmed that WRKY and AP2 TFs were involved in the regulation of the flavonoid biosynthesis in some plants other than *Freesia*. As mentioned above, the expression of WRKY and AP2 family TFs was significantly different in the petals of *Freesia* cultivars with different flower colors. Therefore, to explore more novel TFs that may affect the flavonoid synthesis pathway in the petal of *Freesia*, the WRKY and AP2 TFs and the above structural genes in this pathway were used for cluster analysis based on their FPKM values, and the candidate TFs were screened according to their consistent expression patterns.

**FIGURE 4:**
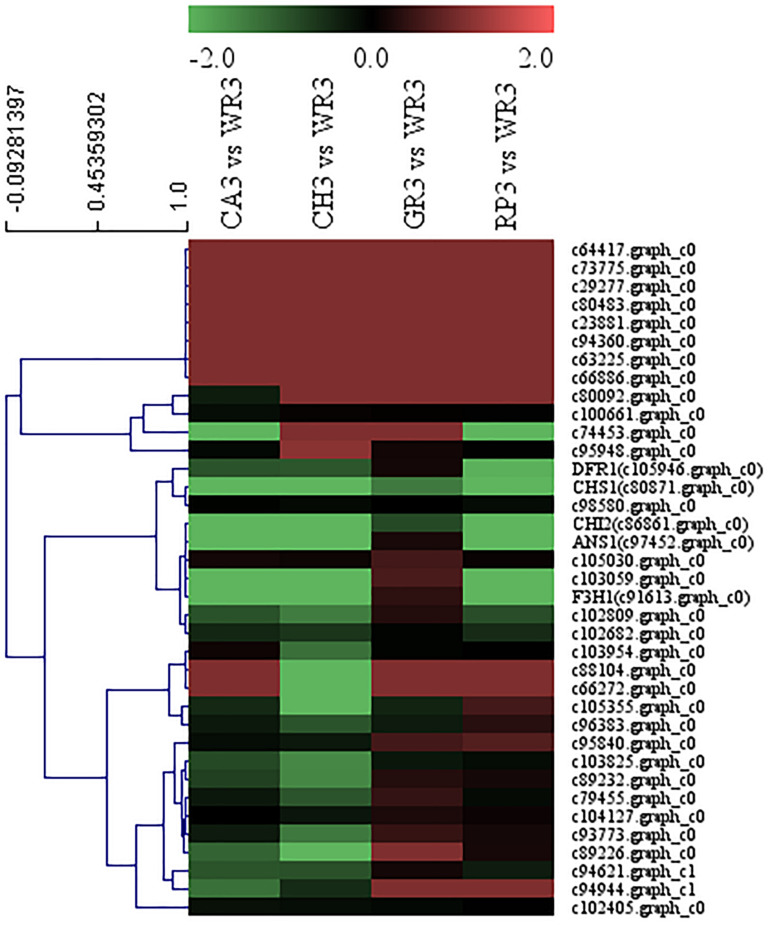
Cluster thermogram of *WRKY* with key structural genes in flavonoid biosynthesis process of *Freesia hybrida*.

A total of 32 WRKY family TFs with five key structural genes involving in the flavonoid biosynthesis pathway in the petals of *Freesia*, namely, *CHS1* (c80871.graph_c0), *CHI2* (c86861.graph_c0), *F3H1* (c91613.graph_c0), *DFR1* (c105946.graph_c0), and *ANS1* (c97452.graph_c0), were combined to perform cluster analysis, and the results are shown in Figure 4.

Among all the WRKY TFs clustered to closer branches with the structural genes, the unigenes c74453.graph_c0 and c103059.graph_c0 were screened due to their closest distance. These two genes were then compared with the *Freesia* corm transcriptome database, and it was found that their expression level in the *Freesia* corms was low. Therefore, the genes c103059.graph_c0 and c74453.graph_c0 were then selected as the candidate unigenes.

**FIGURE 5:**
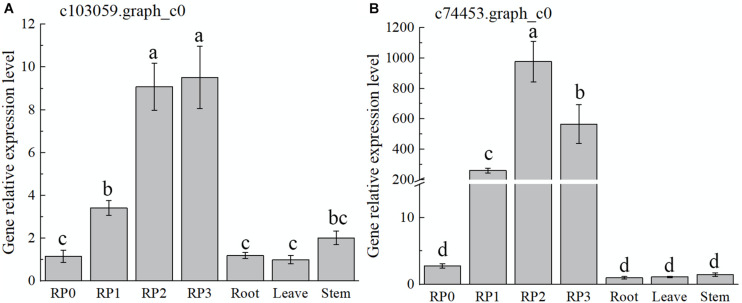
Expression characteristics of two selected *WRKY* TFs in *Freesia hybrida* ‘Red Passion’ (RP). RP0, RP in the green bud stage; RP1, RP in the budding stage; RP2, RP in the early flowering stage; and RP3, RP in the flowering stage. The leaf was chosen as a calibrator sample of c103059.graph_c0 **(A)**. Root was chosen as a calibrator sample of c74453.graph_c0 **(B)**. Lowercase letters stand for the significance of difference at 0.05 level.

As discussed earlier, the highest expression abundance and the highest flavonoid content were observed in the red cultivar RP. Therefore, this cultivar was chosen to verify the temporal and spatial expression characters of selected candidate TFs by using qRT-PCR (Figure 5). The expression of the gene c103059.graph_c0 showed a gradual increase with the development of RP petals, reaching the maximum at S3 (Figure 5A), which was consistent with the accumulation of flavonoids. However, the expression levels of this gene in leaves and stems were much lower than that in petals. With the development of petals, another candidate gene, c74453.graph_c0, showed a slightly different trend, which peaked at S2 and then declined slightly at S3; meanwhile, its expression in roots, leaves, and stems was extremely low (Figure 5B).

**FIGURE 6:**
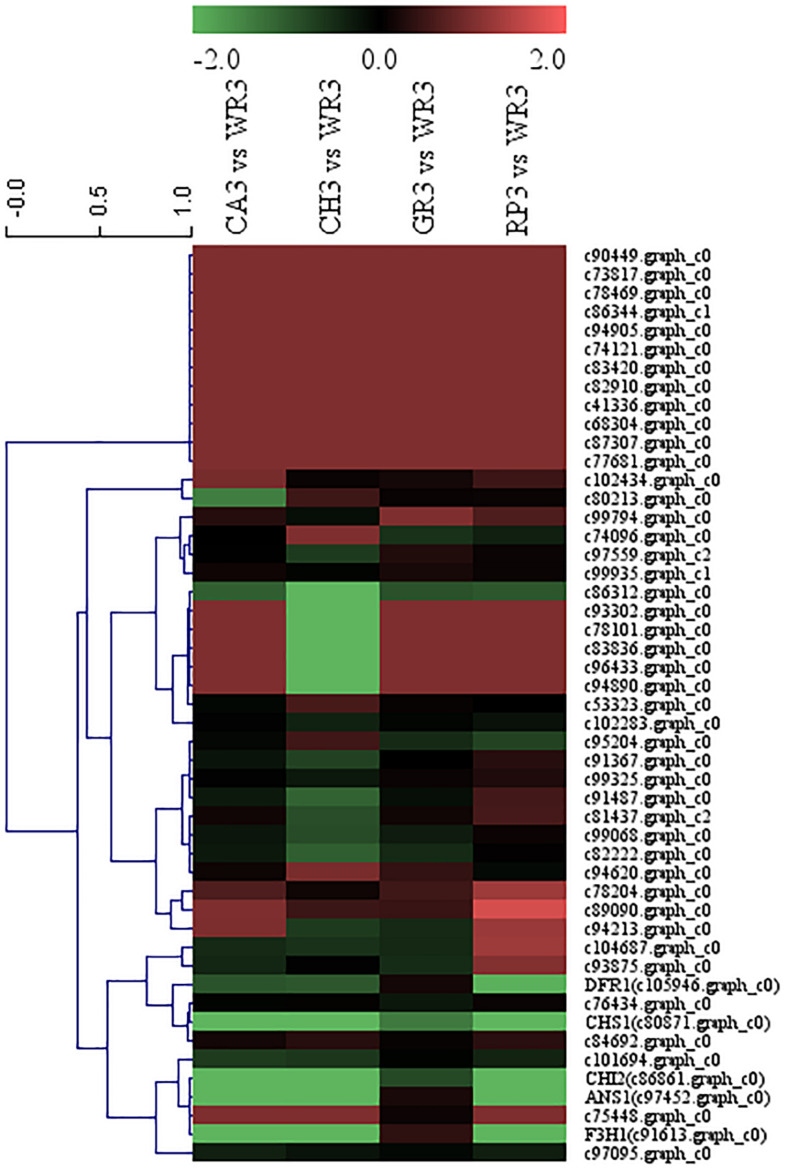
Cluster thermogram of *AP2* with key structural genes in flavonoid biosynthesis process of *Freesia hybrida*.

After preliminary screening, 44 AP2 family TFs were combined with the above five structural genes to perform cluster analysis, and the results are shown in Figure 6. Similar to the above *WRKY* screening, two *AP2* family genes, c97095.graph_c0 and c101694.graph_c0, were chosen and compared with the *Freesia* corm transcriptome database, and the expression levels of the selected two TFs in the *Freesia* corms were also much lower than those in the petals, indicating that these two AP2 TFs had higher specificity in petals. Therefore, these two AP2 TFs were then selected as candidate unigenes to further study their pattern of gene expression of different flowering stages and different tissues by using qRT-PCR in the red cultivar RP.

**FIGURE 7:**
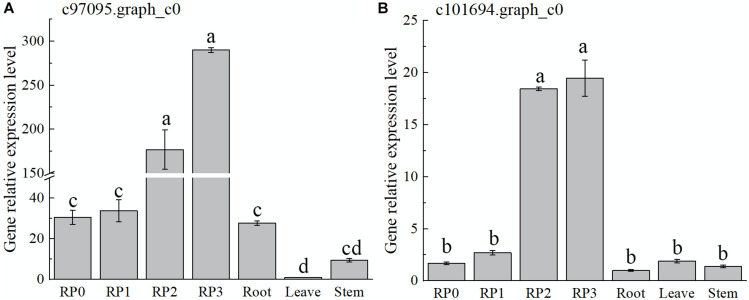
Expression characteristics of two selected *AP2* in *Freesia hybrida* ‘Red Passion’ (RP). RP0, RP in the green bud stage; RP1, RP in budding stage; RP2, RP in early flowering stage; and RP3, RP in flowering stage. The leaf was chosen as a calibrator sample of c97095.graph_c0 **(A)**. Root was chosen as a calibrator sample of c101694.graph_c0 **(B)**. Lowercase letters stand for the significance of difference at 0.05 level.

The expression of both genes, c97095.graph_c0 and c101694.graph_c0, showed a gradual increase with the development of petals and reached the maximum at S3 (Figure 7), which was consistent with the accumulation of flavonoids in the petals of the cultivar. Meanwhile, the expression of these two genes, especially c97095.graph_c0, was significantly higher at S2 and S3 than that at S0 and S1 of petals and other tissues (Figure 7). The spatiotemporal expression pattern showed that these four TFs were expressed in large quantities in petals, revealing that they may play an important role in the regulation of flower color.

## Discussion

### Flavonoid Accumulations Affect Color in *Freesia* Flowers

Four flavonoids and flavonol aglycones, namely, quercetin, kaempferol, isorhamnetin, and naringenin, and a total of 10 anthoxanthin components were detected in *Freesia* petals by UPLC-Q-TOF-MS in this present study. These components have been reported in some ornamental flowers. For example, quercetin, kaempferol, and isorhamnetin were reported in *Primula officinalis* (Karl et al., 1981), and the apigenins, luteolins, and hollandrins were detected in *Iris* × *hollandica* and *Iris rossii* but not reported in *Freesia* (Mizuno et al., 2012, 2013). Kaempferol and quercetin flavonoids were detected in water lilies (*Nymphaea* spp.) and lilies (*Lilium*) and confirmed to affect the appearance of flower color (Guo et al., 2015; Wu et al., 2016). A variety of flavonoids, such as naringenin, quercetin, kaempferol, and luteolin, were also identified in *Zantedeschia hybrida*, which were closely related to the color appearance of yellow, pink, and red calla lily cultivars (Lei et al., 2017).

Anthocyanins were detected in the petals of orange, red, and purple cultivars of *Freesia* but not in white and yellow cultivars. The six most common anthocyanins in plants, namely, cyanidin, delphinidin, petunidin, peonidin, malvidin, and pelargonidin, were all observed in the petals of *Freesia* in this present study. Among them, pelargonidin-3-*O*-glucoside, delphinidin-rutinoside, and cyanidin-triglucoside were first identified in *Freesia*. So far, it is the first proof that *Freesia* contains all the six anthocyanin aglycones.

In orange, yellow, and white cultivars of *Freesia*, the anthoxanthin peaked at S0, followed by a rapid decline, which was similar to the case with *Peony*, *Magnolia biondii*, and *Lonicera* (Yang et al., 2015; Wang et al., 2019; Liu et al., 2020), while in the red and purple *Freesia* cultivars, the anthoxanthin increased first up to S2 and then decreased at S3. Starting from S1, the anthoxanthins in the orange, yellow and white cultivars declined rapidly, while it increased in the red and purple cultivars. It suggested that the anthoxanthins in the petals of orange, yellow, and white cultivars of *Freesia* were synthesized faster than the red and purple cultivars, and the synthesis of the main anthoxanthins was basically completed at S0, while the red and purple cultivars required more anthoxanthins, so they continued to be synthesized at S1 and S2.

During the flower opening in orange, red, and purple cultivars of *Freesia*, anthocyanins showed an upward trend, reaching a maximum at S3, which was significantly higher than that at S0 and S1. This trend was similar to that of *Matthiola incana*, *Primulina swinglei*, and other plants (Hu et al., 2018; Nuraini et al., 2020). In CH, RP, and CA, the anthocyanin content kept a continuous increase from S0 to S3. Up to S3, the accumulation slowed down to some extent. The anthocyanin content in the petals of the red and purple cultivars of *Freesia* was always significantly higher than that of the orange cultivar, and the anthocyanin content between the cultivars was significantly different. These results indicated that the accumulation of anthocyanins in different cultivars was different.

The correlation analysis (Supplementary Table 8) was further conducted based on the flavonoid contents and CIE *L*^∗^ *a*^∗^ *b*^∗^ values of *Freesia* petals (data not shown). The results showed that the more accumulation of flavonoid, the less brightness, and the more redness were possessed in the petals of *Freesia*, revealing that the flavonoid accumulation affected directly the petal color of *Freesia*. It is already clear that the synthesis and accumulation of flavonoids are closely related to the structural genes and TFs in its synthesis pathway. Therefore, to clarify the mechanism of formation of flower color in *Freesia*, it is necessary to further study the expression differences of related genes and explore more novel TFs.

### Expression of Genes Involved in Flavonoids Biosynthesis Is Correlated With the Flower Color of *Freesia*

Based on the above component analysis, it was the first time to detect all three branches of anthocyanin biosynthesis existed in *Freesia*. We further analyzed the gene expression in these pathways.

The structural genes in the flavonoid biosynthesis pathway played an important role in the synthesis of anthoxanthins and anthocyanins. It was already known that genes *CHS*, *CHI*, and *F3H* were the most upstream structural genes in this pathway, affecting the expression of downstream genes and the accumulation of flavonoids. For instance, in pomegranate, it was found that the expression of genes *CHS* and *CHI* was synergistic, and their synergistic expression was beneficial to the synthesis of downstream flavonoids (Zhao and Yuan, 2019). In Yunnan peony (*Paeonia delavayi*), the expression pattern of *PlCHS3*, *PlCHI1*, *PlF3H1*, and other genes in the purple-red cultivar was consistent with the pattern of flavonoid accumulation, which played an important role in the formation of its flower color (Shi et al., 2015). Similarly, in the tulip (*Tulipa fosteriana*), the expression levels of *TfCHS1*, *TfCHI2*, and *TfF3H1* continued to increase as the petal development, leading to the accumulation of anthocyanins and flavonols in the petals, and the color of the flower became darker (Yuan et al., 2014). Studies also showed that the blocked expression of *CHS* in the petals of *Parrya nudicaulis* affected the synthesis of flavonoids and made the petals appear white color (Dick et al., 2011). Our findings showed that in the light-colored *Freesia* cultivars GR and WR, the expression levels of *CHS1*, *CHI2*, and *F3H1* were lower, which may cause lower TFC and lighter color in their petals. Its synergistic upregulated expression can directly affect the accumulation of anthocyanins in the petal development of the dark-colored cultivars of *Freesia*.

*DFR* and *ANS* are the two key genes for anthocyanin synthesis branches. In *Freesia* petals, the expression of *DFR1* and *ANS1* was generally at a high level in the dark-colored cultivars. In contrast, anthocyanins were not detected in yellow and white cultivars (GR and WR). Accordingly, the expression of *DFR1* and *ANS1* in the two cultivars was present a very low level, in contrast to the dark-colored cultivars. The low expression led to the hindrance of anthocyanin synthesis, thereby affecting the appearance of flower color in the yellow and white cultivars. Similar results were reported in *Centaurea cyanus* and roses (*Rosa multiflora*) (Deng et al., 2019; Huang et al., 2019). *FLS*, catalyzing the synthesis of flavonols, competes with the anthocyanin synthesis branch gene *DFR* for the common substrate. The expression of gene *FLS1* in CA and RP was significantly higher than that in other cultivars, which was consistent with the accumulation pattern of anthoxanthin content in the petals of *Freesia*, indicating that the expression of *FLS1* affected anthoxanthin in petals of various cultivars. UF3GT was at the end of the flavonoid biosynthesis pathway, catalyzing the combination of flavonoid aglycone and glycosyl to form a more stable glycoside structure. It was verified that *Fh3GT2* in the *Freesia* red cultivar ‘Red River’ was preferentially involved in glycosylation of kaempferol, while *Fh3GT1* was involved in the glycosylation process of quercetin and anthocyanin (Meng et al., 2019). The expression of *3GT1* selected in this study was significantly higher in the dark-colored cultivars than that in the light-colored cultivars, consistent with the distribution of flavonoids, indicating that this gene had an effect on the glycosylation of flavonoids in the petals of *Freesia*.

### Novel Candidate Transcription Factors Identified From the Transcriptome Database Might Regulate Flavonoids Biosynthesis in *Freesia* Flowers

As already known, some TF families can regulate the structural genes of the flavonoid synthesis pathway. Among them, three TF families, MYB, bHLH, and WD40, were extensively studied, including *Freesia*, and their regulating mechanism was relatively clear. It is natural to consider whether there exist other TFs except the above three TF families, which are involved in regulating the flavonoid synthesis in a certain plant. Recently, it is a hotspot to explore related novel TFs and enrich the mechanism of formation of flower color. So far, there have also been some studies focusing on other TF families related to flavonoid synthesis in some plant species. Among them, some TFs in WRKY, AP2, bZIP, NAC, and other families have been found to be closely related to the anthocyanin synthesis (Rameneni et al., 2020), especially the AP2 and WRKY TF families, which have become research hotspots in recent years. Therefore, some candidate WRKY and AP2 families were selected to explore the possible roles related to the flavonoid biosynthesis in petals of *Freesia*.

**FIGURE 8:**
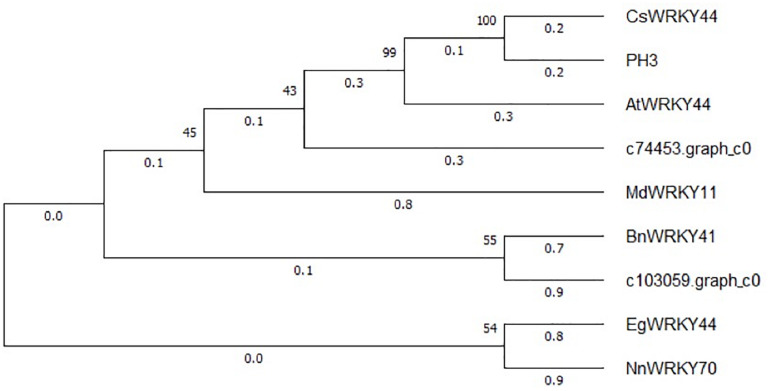
Phylogenetic tree of two selected *WRKY* unigenes and *WRKY* TFs from other plant species. The sequences included *Camellia sinensis CsWRKY44* (MG298960.1), *Petunia hybrida PH3* (AMR43368.1), *Arabidopsis thaliana AtWRKY44* (NM_129282.4), *Malus domestica MdWRKY11*(HM122714.1), *Brassica napus BnWRKY41*(XM_013831080.2), *Elaeis guineensis EgWRKY44*(XM_029264803.1), and *Nelumbo nucifera NnWRKY70*(XM_010253742.2).

**FIGURE 9:**
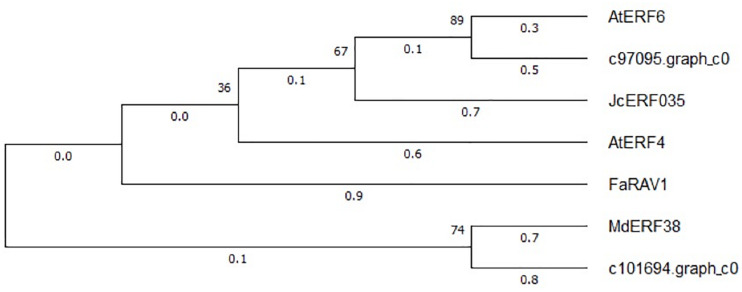
Phylogenetic tree of two selected *AP2* unigenes and *AP2* TFs from other plant species. The sequences included *Arabidopsis thaliana AtEFR6* (AB013301.1), *Jatropha curcas JcERF035* (XM_012237693.3), *Arabidopsis thaliana AtERF4* (NM_112384.2), *Fragaria* × *ananassa FaRAV1* (XM_011466945.1), and *Malus domestica MdEFR38* (MG099847.1).

The expression patterns of the two WRKY TFs selected in this study were consistent with the accumulation pattern of flavonoids in the petals of *Freesia*, thus revealing that they may involve in functions related to flavonoid synthesis. By constructing the phylogenetic tree (Figure 8), we found that these two candidate *WRKY* genes were clustered with *AtWRKY44*, *PH3*, *CsWRKY44*, *MdWRKY11*, and *BnWRKY41*, suggesting that they may have similar functions to these genes. Earlier studies showed that *MdWRKY11* in apples had a positive regulatory effect on the expression of genes such as *F3H*, *DFR*, *ANS*, and *UFGT*, which can affect the synthesis of flavonoids (Wang et al., 2018). *AtWRKY44* in *Arabidopsis* affected the synthesis of proanthocyanidins (Johnson et al., 2002; Gonzalez et al., 2016), and the *PH3* of petunia can bind to *WD40* family TFs, thereby affecting the regulation of anthocyanin synthesis pathways (Faraco et al., 2014; Verweij et al., 2016). In tea plants, the gene *CsWRKY44* plays a key role in regulating the synthesis of catechins (Zhang et al., 2018). Duan et al. (2018) found that *BnWRKY41* in *Brassica napus* affected its anthocyanin accumulation when overexpressed in *Arabidopsis thaliana*. Similar to WRKY TFs, the expression of two selected AP2 TFs was also consistent with the flavonoid accumulation in the *Freesia* petals, and they were clustered with two known *AP2* TFs based on the phylogenetic analysis (Figure 9). In *Jatropha curcas*, the *AP2* family gene *JcERF035* was confirmed to affect the synthesis and accumulation of anthocyanins in its tracheal tissue (Chen Y. et al., 2018). In apples, *MdERF3* was also found to interact with *MdMYB1* to positively regulate anthocyanin synthesis (An et al., 2020). Hence, the candidate *AP2* TFs in *Freesia* may have similar functions to these genes.

In addition, the temporal and spatial expression of these four candidate TFs showed an increasing trend along with the development of flower, and a much higher level was present in petals than other tissues in the red cultivar RP. The above results indicate that the selected WRKY and AP2 family TFs may be involved in the regulation of flavonoid synthesis in the petals of *Freesia*.

## Conclusion

**FIGURE 10:**
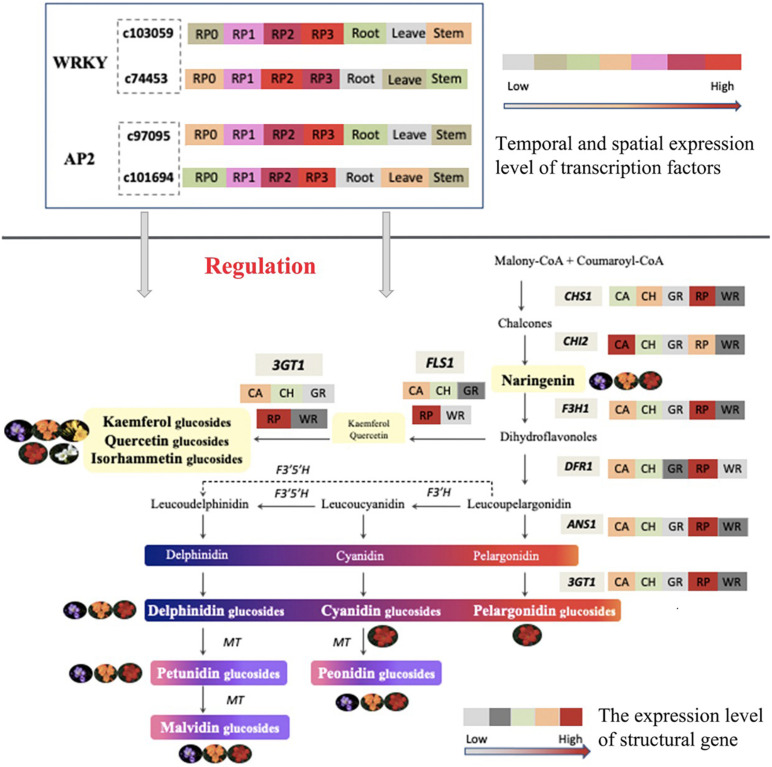
Proposed model of the metabolism and gene regulation of flavonoid in the petals of *Freesia*.

Taken together, the composition and content of anthoxanthin and anthocyanin varied among *Freesia* cultivars with different flower colors and were relative to their flower color. All six common anthocyanin aglycones in plants were detected, and all three anthocyanin biosynthesis branches were proved as existing in *Freesia* at first time in this study. The expression of key structural genes was consistent with the component and accumulation of flavonoids in petals of different *Freesia* cultivars. Combined the transcriptomic and expression analysis, two WAKY TFs and two AP2 TFS were screened, and their spatiotemporal expression characters suggested that these novel candidate TFs may participate in the regulation of flavonoid synthesis in the petals of *Freesia*. A model of flavonoid metabolism and its gene regulation in *Freesia* petals is proposed in Figure 10. The findings of this study laid a solid foundation for the study of molecular mechanism and gene function of the formation of flower color in *Freesia* in future.

## Data Availability Statement

The original contributions presented in the study are publicly available. This data can be found here: National Center for Biotechnology Information (NCBI) BioProject database under accession number PRJNA656641.

## Author Contributions

JZ performed the experiments, analyzed the data, and drafted the manuscript. DT and XG revised the manuscript and helped in completing the final manuscript. XL assisted in the UPLC-Q-TOF-MS analysis. DT designed the experiments, provided guidance on the whole study, and contributed with valuable discussions. All authors contributed to the article and approved the submitted version.

## Conflict of Interest

The authors declare that the research was conducted in the absence of any commercial or financial relationships that could be construed as a potential conflict of interest.

## Publisher’s Note

All claims expressed in this article are solely those of the authors and do not necessarily represent those of their affiliated organizations, or those of the publisher, the editors and the reviewers. Any product that may be evaluated in this article, or claim that may be made by its manufacturer, is not guaranteed or endorsed by the publisher.

## Footnotes

1
http://trinityrnaseq.sourceforge.net/

2
http://www.geneontology.org

3
http://www.genome.jp/kegg

4
https://www.ncbi.nlm.nih.gov/research/cog/

5
http://pfam.sanger.ac.uk/

6
http://www.hmmer.org/

7
http://kobas.cbi.pku.edu.cn/help.do

8
https://sourceforge.net/projects/mev-tm4/

9
http://www.premierbiosoft.com/products/products.html
