# Supplementary material for: Composition of Flavonoids in the Petals of Freesia and Prediction of Four Novel Transcription Factors Involving in Freesia Flavonoid Pathway
Source: Front Plant Sci. 2021 Nov 15;12:756300. doi: 10.3389/fpls.2021.756300 (PMC8634401; doi:10.3389/fpls.2021.756300)
Supplement: Supplementary file 1 [file Data_Sheet_1.zip › Supplementary Figures 1-3 .PDF]

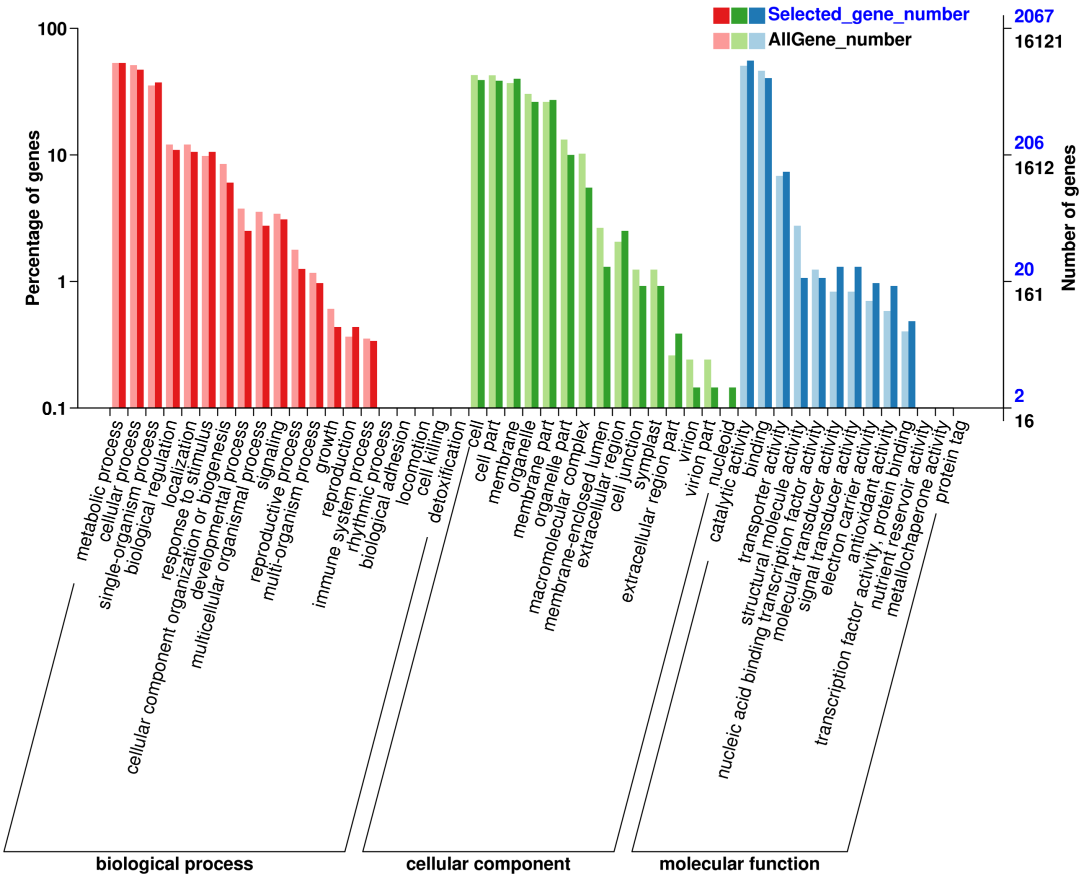

Figure S1. All DEGs annotated with GO classification in CA3 vs WR3, CH3 vs WR3, GR3 vs WR3andRP3 vs WR3

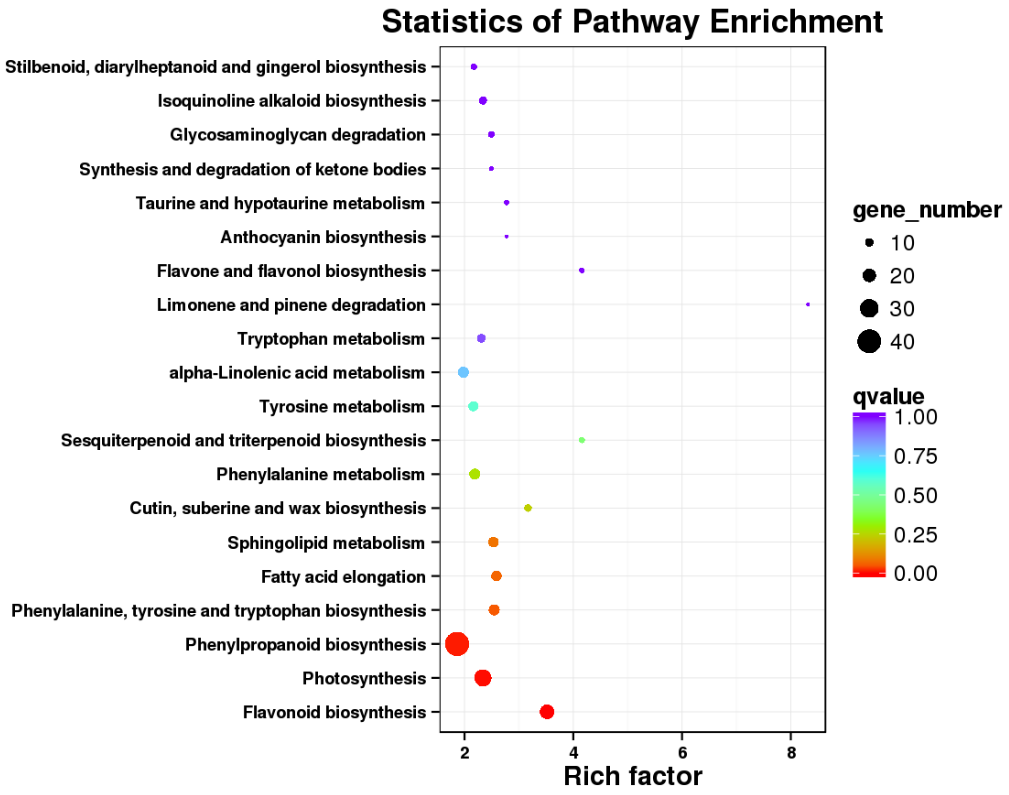

Figure S2. KEGG pathways analysis of All DEGs in CA3 vs WR3,

CH3 vs WR3, GR3 vs WR3 and RP3 vs WR3

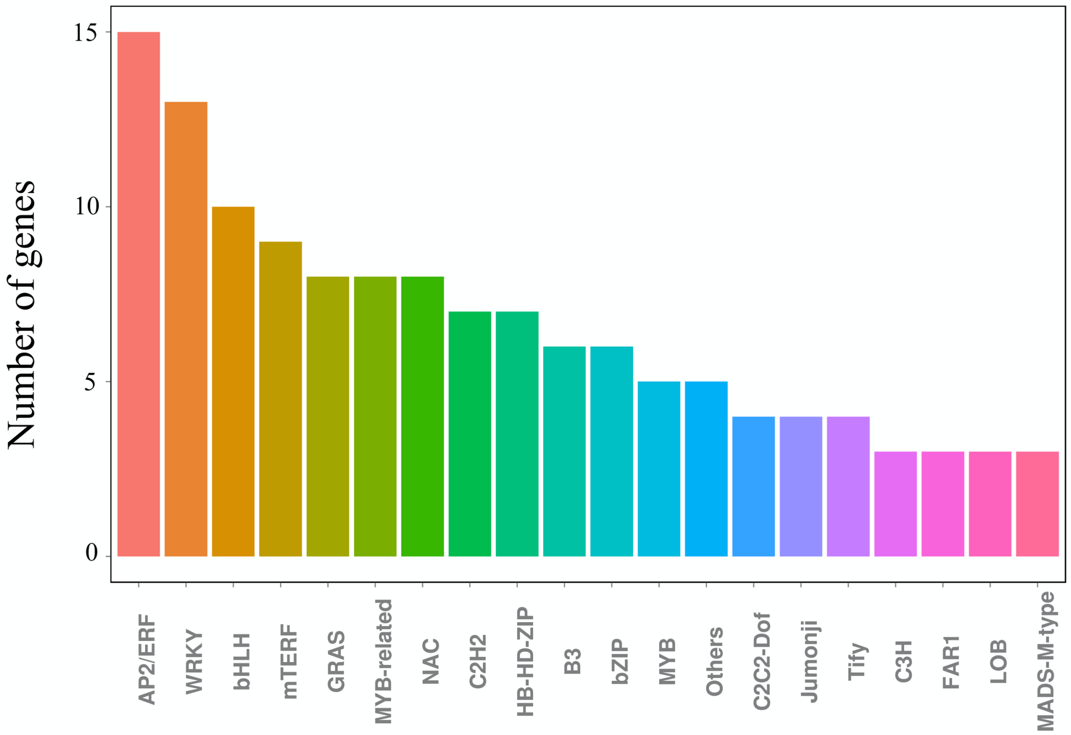

Figure S3. Main differentially expressed transcription factor families in CA3 vs WR3, CH3 vs WR3, GR3 vs WR3 and RP3 vs WR3
