# Supplementary material for: Composition of Flavonoids in the Petals of Freesia and Prediction of Four Novel Transcription Factors Involving in Freesia Flavonoid Pathway
Source: Front Plant Sci. 2021 Nov 15;12:756300. doi: 10.3389/fpls.2021.756300 (PMC8634401; doi:10.3389/fpls.2021.756300)
Supplement: Supplementary file 1 [file Data_Sheet_1.zip › Supplementary Table 1.DOCX]

**Table S1.** Division and description of the 4 flower developmental stages of *Freesia hybrida*

| Developmental stages | Phenotypes of flowers |
| --- | --- |
| The Green Bud Stage (S0) | The flower bud begins to expand, about 1 cm in length |
| The Budding Stage (S1) | The flower bud swells and begins to develop color, about 2.5 cm in length |
| The Early Flowering Stage (S2) | The petals begin to stretch and the flower is about 3.5 cm long, with initial ornamental value |
| The Flowering Stage (S3) | The petals are fully bloomed and the flower is about 4 cm in length, with its color and pattern characteristics fully displayed |
