# Supplementary material for: Composition of Flavonoids in the Petals of Freesia and Prediction of Four Novel Transcription Factors Involving in Freesia Flavonoid Pathway
Source: Front Plant Sci. 2021 Nov 15;12:756300. doi: 10.3389/fpls.2021.756300 (PMC8634401; doi:10.3389/fpls.2021.756300)
Supplement: Supplementary file 1 [file Data_Sheet_1.zip › Supplementary Table 2.DOCX]

**Table S2.** Standards used in UPLC-Q-TOF-MS

| No. | Standard Name | Type | Manufacturer |
| --- | --- | --- | --- |
| 1 | Kaemperide | Anthoxanthin | Shanghai Yuanye Bio-Technology Co.,Shanghai |
| 2 | Genkwanin |  |  |
| 3 | Quercetin 3-O-galactoside |  |  |
| 4 | Myricetin |  |  |
| 5 | Isorhamnetin |  |  |
| 6 | Quercetin |  |  |
| 7 | Kaempferol |  |  |
| 8 | Luteolin | Anthoxanthin | Shanghai Yuanye Bio-Technology Co.,Shanghai |
| 9 | Quercetin 3-O-rutinoside |  | BBI Life Sciences Corporation, Shanghai |
| 10* | European Pharmacopoeia Reference Standard | Anthocyanin | EDQM, France |
| 11 | Pelargonidin 3-O-glucoside |  | Extrasynthese, France |

Note: [10] is mixed standard, including 20 anthocyanin standards such as Malvidin 3-O-glucoside, Delphinidin 3-O-glucoside, Petunidin 3-O-glucoside, Cyanidin 3-O-glucoside, Peonidin 3-O-glucoside, etc.
