# Supplementary material for: Composition of Flavonoids in the Petals of Freesia and Prediction of Four Novel Transcription Factors Involving in Freesia Flavonoid Pathway
Source: Front Plant Sci. 2021 Nov 15;12:756300. doi: 10.3389/fpls.2021.756300 (PMC8634401; doi:10.3389/fpls.2021.756300)
Supplement: Supplementary file 1 [file Data_Sheet_1.zip › Supplementary Table 3.DOCX]

**Table S3.** Primer sequences used in this study

| Gene | Gene Family | Forward Primer(5’ to 3’) | Reverse Primer(5’ to 3’) |
| --- | --- | --- | --- |
| *CHS1* | CHS | CTCTTTTTTGGTCATCAACCCTAC | ATGGACGAGATGAGGAAGAGGT |
| *CHI2* | CHI | ATGGCTTTTCCTATTCGTATGC | CTGACAGTGGCAATCTTGGAGT |
| *F3H1* | F3H | GTTGACTTCTATCCTGGTCCTTGC | TCGCTGAGATGTATCGGAGGAA |
| *ANS1* | ANS | ATGTTGGAGCAAGACAAGGAAA | TTTCGTGGGCTGTCTTCTGTGA |
| *DFR1* | DFR | ACCCCACCAACCTGAGAAAGAC | GTCCCCGCAGATGATGTGAAAA |
| *FLS1* | FLS | CCTAACCTGCTCTTTCTTTTGA | CAACGCCATCATCGTCCACATT |
| *3GT1* | UF3GT | GCTCTAATACCCTTATCTCACTCA | GCGAAGAAGGGAGGAAGATGAGGG |
| *MYB5* | MYB | AGGAGTGAGGGAGGTATGTGAA | TCTCATCCTCAGGCTCCATAAG |
| *GL3L* | bHLH | TCTTGGCTTTTAGGTTGAGTGC | TCTCAAATCACTTGTTCCTTCC |
| *TT8L* | bHLH | AGCCACCGTTGCTTTGGTAATA | GACAAAGATGGATAAAGCCTCG |
| *TTG1* | WD40 | GGGCTTCATTATGCGGCTATTA | TCCATACTTGCTTTCCCCTCAG |
| c103059.graph_c0 | WRKY | TTCTGTCACTTCCCATCCAACC | ACACTAATGTGACGGCAAGGAA |
| c74453.graph_c0 | WRKY | CCAAGGAGTTACTACAGATGCG | AATACAGTTCCGAAACGCCGAG |
| c97095.graph_c0 | AP2 | AGCATAACAGTGGCTCCCTCAT | TTGCTCGCCACCGATAGTTAGA |
| c101694.graph_c0 | AP2 | GGGATTCCACGGCTCACTAAGA | TATGACCACTTGGGTGTTCTTG |
| *Actin* | Actin | CCTCTCTCGGTGA  GGA | TCCAGGCTGTCCTGTC |
