# Supplementary material for: Composition of Flavonoids in the Petals of Freesia and Prediction of Four Novel Transcription Factors Involving in Freesia Flavonoid Pathway
Source: Front Plant Sci. 2021 Nov 15;12:756300. doi: 10.3389/fpls.2021.756300 (PMC8634401; doi:10.3389/fpls.2021.756300)
Supplement: Supplementary file 1 [file Data_Sheet_1.zip › Supplementary Table 4.DOCX]

**Table S4.** Assembly result statistics

| Length range | Transcript | Unigene |
| --- | --- | --- |
| 200-300 bp | 34,308(16.20%) | 26,881(26.74%) |
| 300-500 bp | 42,667(20.14%) | 27,608(27.46%) |
| 500-1,000 bp | 49,889(23.55%) | 21,133(21.02%) |
| 1,000-2,000 bp | 50,251(23.72%) | 14,117(14.04%) |
| 2,000+ bp | 34,695(16.38%) | 10,800(10.74%) |
| Total number | 211,810 | 100,539 |
| Total length | 238,616,790 | 85,738,056 |
| N50 length | 1,796 | 1,523 |
| Mean length | 1,126.56 | 852.78 |

Note：The percentage represents the rate between the number of in corresponding length interval and the total number of Transcripts or Unigenes, respectively.
