# Supplementary material for: Composition of Flavonoids in the Petals of Freesia and Prediction of Four Novel Transcription Factors Involving in Freesia Flavonoid Pathway
Source: Front Plant Sci. 2021 Nov 15;12:756300. doi: 10.3389/fpls.2021.756300 (PMC8634401; doi:10.3389/fpls.2021.756300)
Supplement: Supplementary file 1 [file Data_Sheet_1.zip › Supplementary Table 5.DOCX]

**Table S5.** Statistics of unigene annotation

| Annotated database | Annotated gene number | 300 bp≤Genes≤1,000 bp | Genes≥1,000 bp |
| --- | --- | --- | --- |
| COG annotation | 8,263 | 1,548 | 5,752 |
| GO annotation | 16,121 | 4,833 | 8,976 |
| KEGG annotation | 11,061 | 3,274 | 6,329 |
| KOG annotation | 16,137 | 4,617 | 9,459 |
| Pfam annotation | 18,077 | 4,457 | 11,988 |
| Swissprot annotation | 19,931 | 5,958 | 11,682 |
| EggNOG annotation | 27,486 | 8,701 | 15,064 |
| Nr annotation | 30,090 | 10,106 | 15,698 |
| All annotated | 30,857 | 10,339 | 15,819 |
