# Supplementary material for: Composition of Flavonoids in the Petals of Freesia and Prediction of Four Novel Transcription Factors Involving in Freesia Flavonoid Pathway
Source: Front Plant Sci. 2021 Nov 15;12:756300. doi: 10.3389/fpls.2021.756300 (PMC8634401; doi:10.3389/fpls.2021.756300)
Supplement: Supplementary file 1 [file Data_Sheet_1.zip › Supplementary Table 6.DOCX]

**Table S6.** The number of annotated DEGs in petal transcriptome databases of

5 *Freesia hybrida* cultivars at S3

| DEG Set | DEG Number | Up-regulated DEGs | Down-regulated DEGs |
| --- | --- | --- | --- |
| CA3 vs WR3 | 2,033 | 842 | 1,191 |
| CH3 vs WR3 | 2,584 | 1,039 | 1,545 |
| GR3 vs WR3 | 14 | 6 | 8 |
| RP3 vs WR3 | 2,546 | 1,239 | 1,307 |
| ALL DEGs | 5,162 | 2,254 | 2,908 |

Note：CA3, ‘Castor’ at S3; CH3, SN Chenghuang’ at S3; GR, ‘Gold River’ at S3; RP3, ‘Red Passion’ at S3; WR3, ‘White River’ at S3.
