# Supplementary material for: Composition of Flavonoids in the Petals of Freesia and Prediction of Four Novel Transcription Factors Involving in Freesia Flavonoid Pathway
Source: Front Plant Sci. 2021 Nov 15;12:756300. doi: 10.3389/fpls.2021.756300 (PMC8634401; doi:10.3389/fpls.2021.756300)
Supplement: Supplementary file 1 [file Data_Sheet_1.zip › Supplementary Table 7.DOCX]

**Table S7.** Relevant unigenes involving in the flavonoid biosynthesis of *Freesia hybrida*

| No. | Gene Family | Gene ID | Gene Name |
| --- | --- | --- | --- |
| 1 | CHS | **c80871.graph_c0** | ***CHS1*** |
| 2 |  | c105635.graph_c0 | *CHS2* |
| 3 | CHI | c103498.graph_c1 | *CHI1* |
| 4 |  | **c86861.graph_c0** | ***CHI2*** |
| 5 | F3H | **c91613.graph_c0** | ***F3H1*** |
| 6 |  | c80959.graph_c0 | *F3H2* |
| 7 |  | c76389.graph_c0 | *F3H3* |
| 8 |  | c97564.graph_c0 | *F3H4* |
| 9 | DFR | **c105946.graph_c0** | ***DFR1*** |
| 10 |  | c96436.graph_c0 | *DFR2* |
| 11 | ANS | **c97452.graph_c0** | ***ANS1*** |
| 12 | FLS | **c98090.graph_c0** | ***FLS1*** |
| 13 | UF3GT | **c99634.graph_c0** | ***3GT1*** |

Note: Genes in bold were detected by following qRT-PCR.
