# Supplementary material for: Composition of Flavonoids in the Petals of Freesia and Prediction of Four Novel Transcription Factors Involving in Freesia Flavonoid Pathway
Source: Front Plant Sci. 2021 Nov 15;12:756300. doi: 10.3389/fpls.2021.756300 (PMC8634401; doi:10.3389/fpls.2021.756300)
Supplement: Supplementary file 1 [file Data_Sheet_1.zip › Supplementary Table 8.DOCX]

| CIE L^*^a^*^b^*^ | Pearson Correlation Coefficient | |
| --- | --- | --- |
|  | TFC | TAC |
| *L^*^* | -0.915^**^ | -0.800^**^ |
| *a^*^* | 0.553^*^ | 0.697^**^ |
| *b^*^* | -0.512 | -0.144 |
| *C^*^* | -0.199 | 0.110 |

**Table S8.** Correlation analysis between color parameters and TFC, TAC of *Freesia hybrida*

Note: ^*^means *p*<0.05, ^**^means *p*<0.01.
